# 7th International Multithematic Scientific Bio-Medical Congress (IMBMC), Nicosia, Cyprus, 2019

**DOI:** 10.1038/s41419-020-2300-z

**Published:** 2020-02-04

**Authors:** Panayiota Christodoulou, Panagiotis Boutsikos, Theodora-Christina Kyriakou, Ioannis Patrikios

**Affiliations:** School of Medicine, European University Cyprus, Nicosia, Cyprus

**Keywords:** Biochemistry, Diseases

The 7th International Multithematic Bio-Medical Congress 2019, “Bio-Medical Scientific Cyprus (BSC)”, was organized by the European University Cyprus (EUC), School of Medicine, Nicosia, Cyprus, with the general title: “Medical School: Resource of Science and Culture”; under the auspices of the Ministry of Health and the Cyprus Medical Association (CyMA). BSC is an internationally recognized annual event that was first founded and established by Professor Dr. Ioannis Patrikios, the chairperson and Faculty member of the School of Medicine at EUC.

The keynote speaker, Distinguished Professor Dr. Sir Magdi Yacoub gave a lecture about the investigation of truth in science from ancient years to nowadays. As he pointed out, despite the fact that the science evolves and provides useful tools in humanity, there are many risks. His lecture focused on three main pillars: organ transplantation, tissue regeneration, and tissue engineering. “The main threats that the research faces are false scientific discoveries and the use of finding in a speculative manner” as he further discussed. In addition, the arrogant comprehension and scientific competition set the glory of science in a position of jeopardy as he noted. Clinicians, international organizations, and governments should help the science remain intact and safe as he strongly emphasized.

Professor Dr. Constantine A. Stratakis presented a talk entitled “Discovering New Genetic Syndromes Seeing Patients: Carney-Stratakis Syndrome, 3PAS, iMAD, X-LAG and Other”. He emphasized that discovery contains observing and thinking. Constantine Stratakis and his team identified the majority of genes that are responsible for adrenocortical tumor and pituitary adenoma. The discovering of specific genes led in describing of diseases like Carney-Stratakis syndrome (after his name), 3PAS, iMAD and XLag. Behind the vast majority of disorders there is a well described genetic and molecular background that it is mandatory for the clinicians to take in consideration in order to move on, into the so-called personalized medicine, as he emphasized in his second lecture.

Professor Dr. Isaac Yaniv spoke on neuroblastoma, which is an extracranial childhood malignancy. The therapeutic approaches for neuroblastoma include chemotherapy, radiotherapy, stem cell usage, and isotretinoin. The observation of overexpression of GD2 in cancer cells Vs normal cells, led to the development of a new therapeutic strategy by the use of chimeric antibodies (against GD2) that can selectively target the neuroblastoma cells. The monoclonal antibody therapy increased the survival of patients in clinical trials and indicated better outcome Vs other therapies. In May 2017, the antibodies took market authorization. He further explained that active and passive immunotherapies are under investigation and the response is deferent among patients.

Dr. Samih Al-Hayek discussed novelties in urology, highlighting that although the genitourinary diseases have been recognized for thousands of years, Urology as a specialty was only established in the 1890s. Urological tumors have now become one of the leading causes of cancer death in the male population. We now have better imaging technology with 3D reconstruction allowing precision in diagnosing urological conditions such as prostate cancer, he emphasized. Miniaturization of instruments led to safer operations, quicker recover, and less complications especially in managing renal and ureteric stones. Penile and urethral implants improved patients’ quality of life and their social interaction. More advances are on the horizon such as gene therapy, nanotechonology, and tissue engineering, as he pointed out.

Professor Dr. Sherif Mourad analyzed one subtype of female’s fistula, the Vesicovaginal fistula (VVF) that is a common urogenital problem in developing countries. The main cause as he explained is the “obstructive labor” and includes early pregnancies, restriction in gynecologist observation, malnutrition, female circumcision, and insertion of caustic substances into the vagina with the intent to treat a gynecologic condition or to help the vagina to return to its nulliparous state. Furthermore, prolonged compression of vaginal tissues bladder base and urethra by embryonic head may produces tissue hypoxia and necrosis. In addition, urethral sphincter incompetency and neuropathic bladder disfunction are common complications. VVFs in the developed countries are attributed predominantly to inadvertent bladder injury during pelvic surgery (85%). As Dr. Mourad concluded, the treatment complexity and success depends on multiple factors including: Fistula type, size, degree of scarring, involvement urethra, ureter and bladder, provider capacity, postoperative care and compliance.

Professor Dr. Hans Lassmann presented a talk entitled “Not all is multiple sclerosis (MS), What Has Been Thought to Be: A Comparison between Myelin oligodendrocyte glycoprotein (MOG) Antibody Associated Disease and MS”. MOG antibody associated disease (MOGAbD) and MS are both due to a chronic inflammatory response in the brain, which is associated with focal primary demyelination, axonal preservation and reactive astrocytic gliosis, thus fulfilling the essential criteria for a diagnosis of MS, as he discussed. However, recent data show that these diseases differ in clinical presentation and response to therapy. More recently, detailed information on the pathology of MOGAbD became available that also shows differences to MS. As Professor Lassmann concluded, the domination of brain inflammation and the occurrence of demyelination between these diseases are suggesting that MS and MOGAbD are two distinctly different diseases with fundamentally different pathogenesis.

Professor Dr. Ido Wolf gave a talk entitled “Cannabis for the use against cancer-related symptoms: what do the data teach us?”, and he described current data regarding the efficacy of cannabis in various cancer-related symptoms. The cannabinoids mediate their action through two kinds of G-protein-coupled receptors, CB1 and CB2, found in membranes of nerve cells as he pointed out. The CB1 receptor is found predominantly in the central and peripheral nervous systems and suppresses neuronal excitability, whereas the CB2 receptor is found predominantly in immune tissue and is not related to psychoactive effects. Despite its extensive use, only few controlled trials have tested the efficacy of either synthetic or purified cannabinoids in the treatment of cancer-related symptoms, with most showing negative results. In his final statement Professor Ido emphasized that recent Phase III trials noted a modest or no effect of either THC/CBD extract or the synthetic cannabinoid nabilone compared with placebo for the treatment of intractable cancer-related pain. But, despite lack of evidence-based data, native cannabis use has been legalized in several Western countries, including Israel, and is currently the most commonly prescribed drug for Israeli cancer patients.

Professor Dr. Filippos Triposkiadis lectured on randomized clinical trials using heart failure (HF) patients with low left ventricular ejection fraction (LVEF) to enhance statistical power. However, this use of LVEF in clinical trials has led to oversimplification of the scientific view of a complex syndrome as he explained. HF is a heterogeneous syndrome in which disease progression is associated with a dynamic evolution of functional and structural changes leading to unique disease trajectories creating a spectrum of phenotypes with overlapping and distinct characteristics, as he emphasized.

Professor Dr. Konstantinos Toutouzas presented a talk entitled “Advances in transcatheter implantation of an aortic valve prosthesis (TAVI) 2019”. Symptomatic Severe Aortic Stenosis requires Aortic Valve Replacement, while medical therapy offers only temporary symptomatic relief as he pointed out. In 2002 the first percutaneous TAVI in a patient with calcific aortic stenosis performed and since then TAVI has been established as an additional choice of intervention in patients with severe Aortic Stenosis.

Professor Dr. Miltos Matsangas discussed the findings of the COMPASS- PAD (Cardiovascular Outcomes for People Using Anticoagulation Strategies- Peripheral Arterial disease) trial. It was found that low-dose rivaroxaban (2.5 mg), taken twice a day on top of aspirin (100 mg) once a day reduced death, major adverse cardiovascular and limb events when compared with aspirin alone. However, although fatal or critical organ bleeding was not increased, major bleeding was significantly higher, arising concerns about the widespread usage of this combination in all PAD patients. On the other hand, on specific high-risk patients, the absolute risk of severe bleeding remains acceptable and the net clinical benefit is favorable for the combination of low-dose rivaroxaban and aspirin.

Professor Dr. Eleni Arnaoutoglou gave a talk about perioperative management of Direct Oral Anti-Coagulants (DOACs) discussing the results of a PAUSE study that establishes a new strategy. The study was designed in order to assess that a simple, pharmacokinetic based approach strategy (1 day off before and after procedures with a low risk of bleeding and for 2 days off before and after procedures with a higher bleeding risk) that forgoes heparin bridging and preoperative coagulation testing—is associated with low rates of major bleeding (<2%) and arterial thromboembolism (<1%). Pause study showed that patients with Atrial Fibrillation (AF) who had DOAC therapy interruption for elective surgery or procedure, a perioperative management strategy without heparin bridging or coagulation function testing was associated with low rates of major bleeding and arterial thromboembolism.

Associate Professor Dr. Andreas Pittaras spoke on diagnostic approache of hypertensive patients and discussed the major key steps in physical examination, including several routine laboratory tests as Haemoglobin and haematocrit, Fasting blood glucose and glycated HbA1c, Blood lipids, Potassium and Sodium, Uric acid, Creatinine and eGFR, Liver function tests, Urine analysis (including urinary protein) and 12-lead ECG. Hypertension is predominantly an asymptomatic condition that is best detected by structured population screening programmes or opportunistic measurement of blood pressure (BP). He highlighted that all adults should have their BP recorded in their medical record and be aware of their BP, and further screening should be undertaken at regular intervals with the frequency dependent on the BP level.

Professor Dr. George S. Stergiou gave a talk about the diagnosis and treatment of white-coat and masked hypertension. The evaluation of blood pressure (BP) in the office and out-of-office (self-home or 24-h ambulatory monitoring) identifies individuals with White-coat hypertension, who have elevated BP only in the office (low ambulatory or home BP), and with Masked hypertension, who have non-elevated BP in the office but elevated BP out of the office (ambulatory or home). About 10–30% of subjects have white-coat hypertension and 10–15% have masked hypertension. Professor Stergiou concluded that subjects with Masked Hypertension are at high risk of cardiovascular events as sustained hypertensives and require drug treatment aiming to normalize out-of-office BP.

Dr. Aris Anastasakis discussed the use of genetics in the prevention of sudden cardiac death in the young and athletes. He pointed out that rare diseases of the heart are mainly hereditary diseases and are predominantly related with the phenomenon of sudden death in the young. Therefore, prevention of sudden death in the young, is closely related with the adopted mechanisms, in each country, for early diagnosis and treatment of hereditary cardiovascular diseases as he concluded.

In this meeting report, we have summarized the major scientific findings from the aforementioned presenters and their respective topics. We note that unpublished data are also included.

